# Inscription of Quasi-Sinusoidal Surface Relief Optical Gratings in ZEONOR™ Cyclic Olefin Polymer by a 10.5 MeV N^4+^ Ion Microbeam at Low Implanted Fluences

**DOI:** 10.3390/s26154974

**Published:** 2026-08-05

**Authors:** István Bányász, Gyula Nagy, István Rajta, Vladimír Havránek, András József Laki, Miklós Kellermayer, Ágnes Nagyné Szokol, Robert Magnusson

**Affiliations:** 1 Department of Mathematics and Physics, Széchenyi István University, H-9026 Győr, Hungary; 2 Institute of Applied Physics, TU Wien, 1040 Vienna, Austria; gyulanagy@atomki.hu; 3 Atomki, HUN-REN Institute for Nuclear Research, P.O. Box 51, H-4001 Debrecen, Hungary; rajta@atomki.hu; 4 Nuclear Physics Institute AV CR, 250 68 Řež, Czech Republic; havranek@ujf.cas.cz; 5 Faculty of Information Technology and Bionics, Pázmány Péter Catholic University, Práter u. 50a, H-1083 Budapest, Hungary; laki.andras.jozsef@itk.ppke.hu; 6 Department of Biophysics and Radiation Biology, Semmelweis University, Tűzoltó u. 37-47, H-1094 Budapest, Hungary; kellermayer.miklos@semmelweis.hu; 7 HUN-REN Wigner Research Centre for Physics, P.O. Box 49, H-1525 Budapest, Hungary; szokol.agnes@wigner.hun-ren.hu; 8 Department of Electrical Engineering, University of Texas at Arlington, Arlington, TX 76019, USA; magnusson@uta.edu

**Keywords:** optical micro- and nanostructures, optical grating, cyclic olefin polymer, ZEONOR™, ion microbeam implantation

## Abstract

Direct writing of microstructures in ZEONOR™ cyclic olefin polymer, using a high-energy medium-mass ion microbeam, was proposed and demonstrated. Microbeams of 10.5 MeV N^4+^ ions were used. The implanted fluences were between 2.5·10^12^ and 2.0·10^13^ ion/cm^2^. The microstructures were surface relief optical gratings of quasi-sinusoidal profile and grating constant of 4, 8 and 16 µm. Peak-to-peak amplitudes of the gratings ranged from 10 to 90 nm. Measured profiles of the gratings were Fourier-analyzed. Highest measured net first-order diffraction efficiencies were between 25 and 29% at wavelengths of 405 and 640 nm, very close to the theoretical maximum of 33%, calculated with the Raman-Nath equation. This method could be used for the production of micro- and nanostructures in cyclic olefin polymers and copolymers and other polymers as well, either for the direct production of label-free biosensors and other devices, or to produce masters for hot stamping of such structures.

## 1. Introduction

Cyclic olefin copolymers and polymers, like ZEONOR™, are extensively used for microfluidic devices and microsystems. There is ample literature on polymeric microfluidic devices and microsystems, including some recent review articles [1,2,3,4,5]. Various microelectronic technologies were adapted to the fabrication of micro- and nanostructures in polymers [1,4]. The two main categories are replication methods and rapid prototyping. Hot embossing, injection molding and nanoimprint lithography are the most common methods belonging to the first category. The main methods for direct structuring of the polymer surface are micromachining or micromilling [6], laser ablation [7,8,9] and xurography [10].

Effects of ionizing radiation on polymers have been studied for a long time. Kumar and Adams studied the damage caused to polymer samples by the electron beam in an electron microscope [11]. They studied the structural changes in the polymers induced by electron beam irradiation. Structural parameters were determined from electron diffraction measurements. They came to the conclusion that polymers with high melting temperature remained stable and the critical electron dose was proportional to the melting temperature of the polymer. Lee et al. implanted Kapton, Teflon, Tefzel and Mylar polymers with He, B, C, N and Fe ions in the 0.2–2 MeV energy range [12]. The implanted dose was set to 3·10^15^ ions/cm^2^. They measured the composition of the implanted layers by Rutherford backscattering (RBS). Hardness and wear of the pristine and implanted samples were also measured. The implantation induced a significant improvement in the hardness of the samples, except for 300 keV N-ion-implanted Teflon. The highest increase in hardness, 26-fold, was measured in Kapton, when implanted simultaneously by B, N and C ions. A very detailed review on the effects of ionizing radiation in polymers was published by Ashfaq et al. [13].

The micro ion beam used in the present work was around the lower limit of the so-called swift heavy ions (SHI). There is no strict definition of the SHI, since the ion-matter interaction also depends on the target composition. It is considered that if the kinetic energy per nucleon is over 1 MeV/amu, the ion beam is of SHI. In our experiments, it was 0.75 MeV/amu. Balanzat et al. studied swift heavy ion implantation of polymers [14]. The targets were polyethylene (PE), polyvinylidene fluoride (PVDF) and polystyrene (PS). They used a large number of ion species from C to Pb, with a few MeV/amu specific energy. They found that structural changes induced by SHI implantation manifest themselves clearly in the changes in the near-infrared (NIR) absorption spectra of the polymers. They observed complete or partial destruction of the polymer structure, creation of in-chain saturations, various types of chain scissions, and amorphization. Banerjee et al. implanted polyaniline (PAni) nanofibers with 90 MeV O^7+^ ions at fluences in the range of 3·10^10^–1·10^12^ ions/cm^2^ [15]. They performed various measurements on the pristine and implanted samples. Micro-Raman (µR) studies confirmed amorphization of the PAni nanofibers and also showed that the PAni nanofibers get de-doped upon SHI irradiation. Komarov published a review on nano- and microstructuring of solids by swift heavy ions [16]. A very detailed theoretical description was given on the ion track formation in polymers, based on the modified version of the thermal spike model of Szenes [17]. Samra et al. [18] studied the effects of swift heavy ions on the structural and optical parameters of low-density Polyethylene samples. They used 50 MeV copper and 70 MeV carbon ions. Apel [19] published a detailed theoretical review on the effects of swift heavy ion implantation on polymers. Negi [20] used swift heavy ion implantation with 170 MeV Xe ions to create nanochannels in polyethylene terephthalate (PET) membranes. Another review on the use of swift heavy ions in the modification of polymers was published by Kaur [21]. That work dealt only with ion track nanotechnology and cosmic ray radiation simulation. Saini et al. [22] summarized recent research on engineering of nanochannels in polymer membranes for energy and biological applications, including the use of swift heavy ion implantation. Aubrecht and coworkers [23] fabricated micropatterns in TOPAS™, a Cyclic Olefin Copolymer (COC), using middle-energy ion beams. The material they used was very similar to the ZEONOR™ used in our present research.

Determination of the effects of ion beam implantation on the target material depends on a number of factors, including the material composition, ion species, energy, implanted fluence and current density during implantation. If the target material is a polymer, then that task is even more complicated. Various competing physical and/or chemical processes form the surface structure, such as chain scission and cross-linking in polymers [24,25]. For instance, compaction of polydimethylsiloxane (PDMS) has been observed over a wide range of ion fluence when irradiated with both light [26] and medium-heavy [27] ions. In contrast to PDMS, in poly(methyl methacrylate) (PMMA), swift heavy ion irradiation can induce both swelling and compaction, depending on the applied fluence and the scanning speed of the beam [28]. The structural changes in PMMA are also fluence-dependent. While at low fluences chain scissioning is the dominant process, at sufficiently high fluences cross-linking dominates [29]. Generally speaking, *a priori* determination of the ion beam-induced exact volume changes is not possible because of the lack of knowledge of the exact physical and chemical processes for a given polymer target. Likewise, one cannot always predict the distribution of refractive index change induced in the material.

Direct writing of picowell arrays in ZEONOR™ polymer was proposed and realized by Bányász et al. [30]. A microbeam of 10.5 MeV N^4+^ ions was used, the same as in the actual work. The beam current was 800 pA, and the beam size was 2.5 × 2.5 µm^2^. The ion microbeam induced considerable swelling of the polymer surface. Both disk- and ring-shaped areas of the sample were implanted, as it was not known *ab ovo* whether the implantation would produce swelling or compaction. Implantation of the ring-shaped areas resulted in the creation of tori of a medium diameter of 20 µm and a height of 4.7 µm. Thus, an array of picowells of 820 femtoliter volume was fabricated. The implanted fluence was 7.8·10^12^ ion/cm^2^.

Surface relief transmission optical gratings of quasi-sinusoidal profile and high diffraction efficiency were fabricated in optical glasses and crystals by ion microbeam implantation by Bányász et al. [31,32]. In the first experiment [31], gratings with a 30 μm grating constant were manufactured in glass samples using 5 MeV N^3+^ and 6 MeV O^4+^ ions. Implanted fluences were in the 10^15^–10^16^ ion/cm^2^ range. The gratings were characterized by interference microscopy and microprofilometry. The obtained surface profiles of the gratings were found to be quasi-sinusoidal with up to 265 nm amplitude. Measured highest first-order diffraction efficiencies were around 26% in both Pyrex and IOG glasses. In the second experiment [32], 5 MeV N^3+^ and 10.5 MeV N^4+^ ion microbeams were used to write surface relief transmission optical gratings of quasi-sinusoidal and sawtooth profiles in Bi_12_GeO_20_, Er: LiNbO_3_ and Er:Fe:LiNbO_3_ crystals. Grating constants were 4 and 16 µm. Implanted fluences were between 1·10^14^ and 5·10^15^ ion-cm^2^. Highest measured first-order diffraction efficiencies were around 25%. The highest possible diffraction efficiency of 33% was not attained simply because the quotient of the fluence series was not small enough. Fourier analysis of the quasi-sinusoidal profiles of all ion microbeam-implanted gratings was also performed [33].

## 2. Materials and Methods

**Figure 1:**
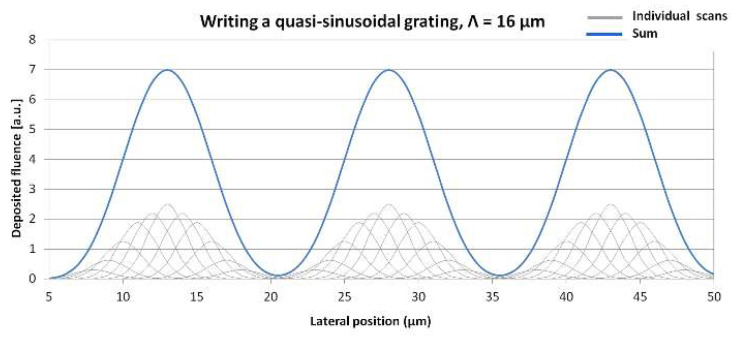
Distribution of the deposited fluence along a sinusoidal grating of a grating constant of 16 μm. The profile of the ion microbeam was approximated by a Gaussian with a FWHM of 2.5 μm. Fluence distributions of the individual scans are represented by gray lines. Individual scans were separated by 1 μm. The sum of the deposited fluence distributions of the individual scans is represented by the blue line. This figure was originally published by Bányász et al. under a CC-BY 4.0 license [31].

Based on the above-mentioned previous results, inscription of surface relief transmission optical gratings of quasi-sinusoidal profile in ZEONOR™ polymer, using a 10.5 MeV energy N^4+^ ion beam, was devised. The polymer was provided by Microfluidic ChipShop (Jena, Germany). As was reported earlier, ion microbeam implantation induced swelling of the implanted surface in that polymer, and the amplitude of the swelling was proportional to the implanted fluence in a wide range. This behavior permitted the control of the profile of the ion microbeam implanted surface structures by spatial modulation of the implanted fluence [31]. Periodic sinusoidal spatial modulation of the implanted fluence resulted in surface relief gratings of quasi-sinusoidal profile [31,32,33]. The method is explained in Figure 1.

The implantations were performed at the Tandetron laboratory of the ÚJV research institute in Řež, Czech Republic. The target material was ZEONOR™ cyclic olefin polymer, in the format of a microscopic slide. It was purchased from Microfluidic ChipShop (Jena, Germany). It was prepared from the mcs-COP 02 version of the ZEONOR™. According to the manufacturer, its glass transition temperature (T_g_) was 136 °C.

The microbeam consisted of N^4+^ ions of 10.5 MeV energy. The cross section of the microbeam on the sample was 2.5 µm × 2.5 µm, and the beam current was 125 pA. Implanted fluences were between 2.5·10^12^ and 4.0·10^13^ ion/cm^2^. Lateral dimension of the implanted optical gratings was 0.5 mm × 0.5 mm. Gratings with grating constants of 4, 8 and 16 µm were written in the sample by magnetic microbeam scan.

## 3. Results and Discussion

**Figure 2:**
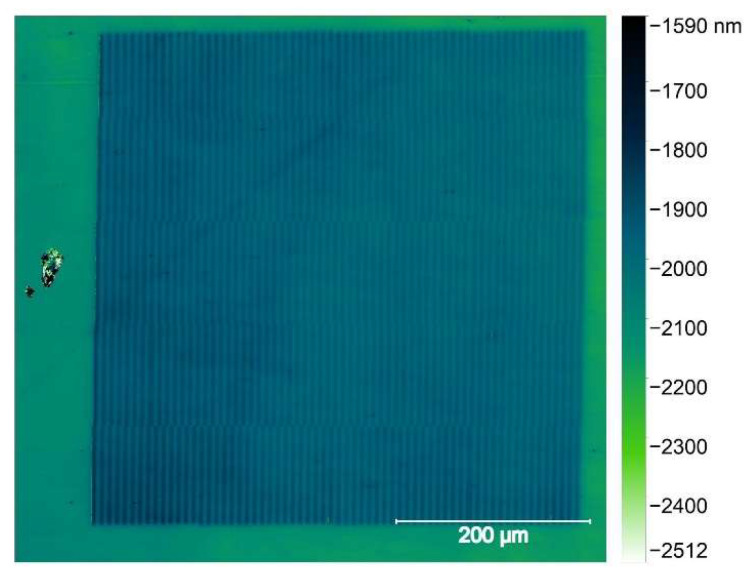
Surface topography of an ion microbeam implanted grating, measured by Zygo NewView 7100. Grating constant Λ = 8 µm. The implanted fluence was 5·10^12^ ion/cm^2^. Surface height is represented in truncated linear false colors. The height scale is on the left of the figure.

The implanted gratings were observed under a Zeiss Peraval microscope (VEB Carl Zeiss Jena, Jena, Germany) in INTERPHAKO mode. Then the complete surface topography of each grating was measured by a Zygo NewView 7100 optical surface profiler (Zygo Corporation, Middlefield, CT, USA) [34]. Surface topography of the gratings was analyzed by the free code Gwyddion [35]. Peak-to-peak amplitude of the gratings was determined by fitting a sine function to sections of the grating surface map parallel to the grating vector. Then gratings were Fourier-analyzed, and amplitudes of the harmonics were determined [27]. Surface topography of an ion microbeam-written grating is presented in Figure 2.

Grating lines are clearly discernible in Figure 2. A slight variation of a in a few tens of nanometers in the average surface height can be attributed to three possible causes: an uncompensated small tilt of the sample during measurement, the stitching process of a number of smaller surface maps, and, eventually, a small local unevenness of the surface of the pristine sample. However, there are only small variations in the peak-to-peak amplitude across the grating.

**Figure 3:**
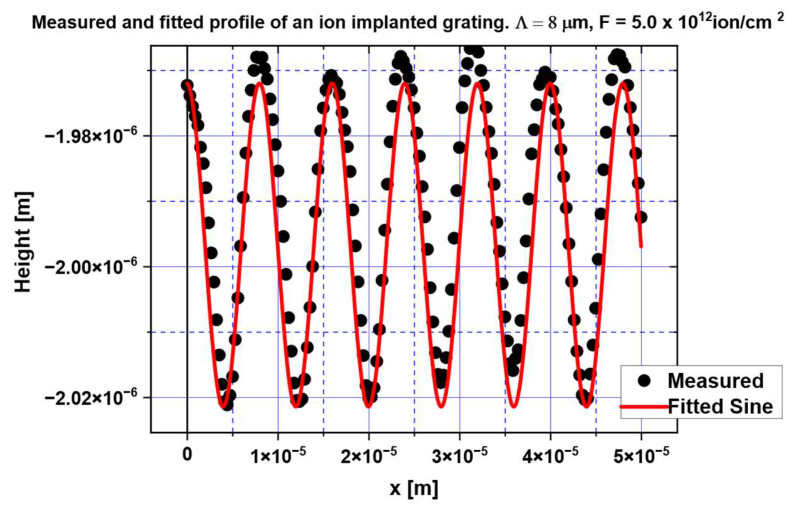
Measured profile of the grating along a line perpendicular to the grating lines (black dots) and a sine curve fitted to the experimental data (red line). Grating constant Λ = 8 µm. The implanted fluence was 5·10^12^ ion/cm^2^.

The measured profile of the same grating along a 50 µm long line, perpendicular to the grating lines, is shown in Figure 3. A biased sine function was fitted to the profile. It can be seen that the grating profile is quasi-sinusoidal.

**Figure 4:**
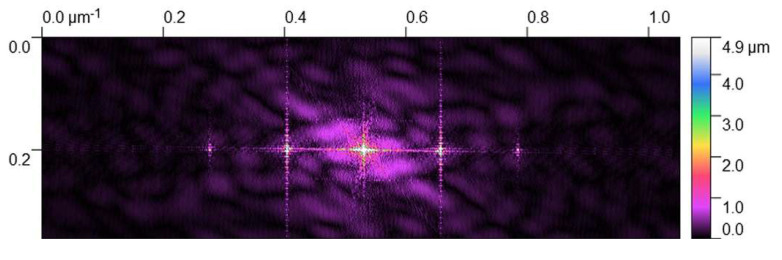
Modulus of the 2-D Fourier transform of the surface topography. Note the peaks along the horizontal axis of the rectangle. They correspond to the harmonics of the grating profile. Grating constant Λ = 8 µm. The implanted fluence was 5·10^12^ ion/cm^2^.

**Figure 5:**
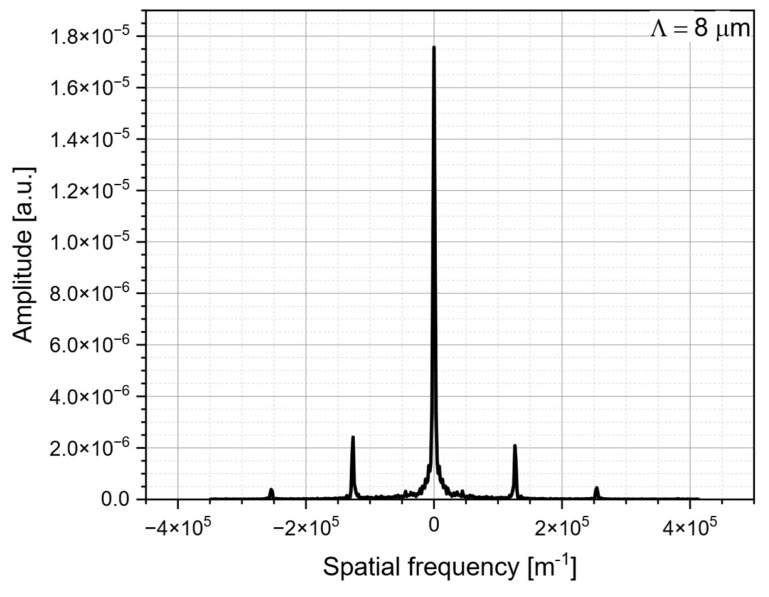
Line profile along the horizontal axis of the Fourier transform. Peaks corresponding to the ±1st and ±2nd harmonics can be observed clearly. Grating constant Λ = 8 µm. The implanted fluence was 5·10^12^ ion/cm^2^.

Results of the Fourier analysis of the same grating are presented in Figure 4 and Figure 5. The central part of the modulus of the 2-D Fourier transform of the surface topography of Figure 2 is presented in Figure 4. The peaks located along the horizontal symmetry axis of the rectangle correspond to the harmonics of the grating profile.

A line profile along the horizontal symmetry axis of Figure 4 can be seen in Figure 5. Peaks corresponding to the 0th, ±1st and ±2nd harmonics can be clearly observed. Spatial frequency is 1.26317·10^5^ m^−1^, corresponding to Λ = 7.94 µm.

The amplitudes of the harmonics of the grating were determined from the 2-D Fourier transform of the surface topographies.

**Figure 6:**
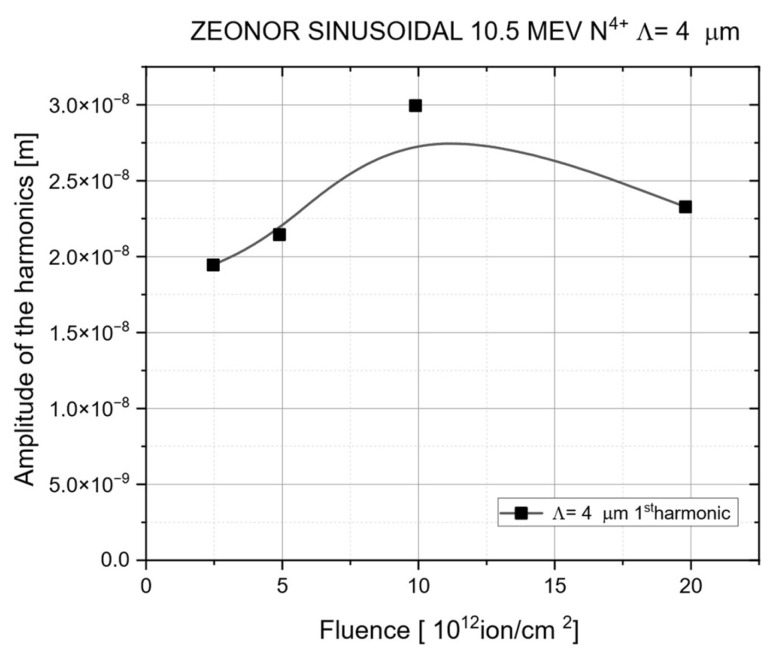
Amplitudes of the harmonics of the gratings implanted in ZEONOR™ by 10.5 MeV N^4+^ ions vs. implanted fluence. Λ = 4 μm. Black squares: measured values of the amplitude of the first harmonic. The black line serves only to guide the eye.

**Figure 7:**
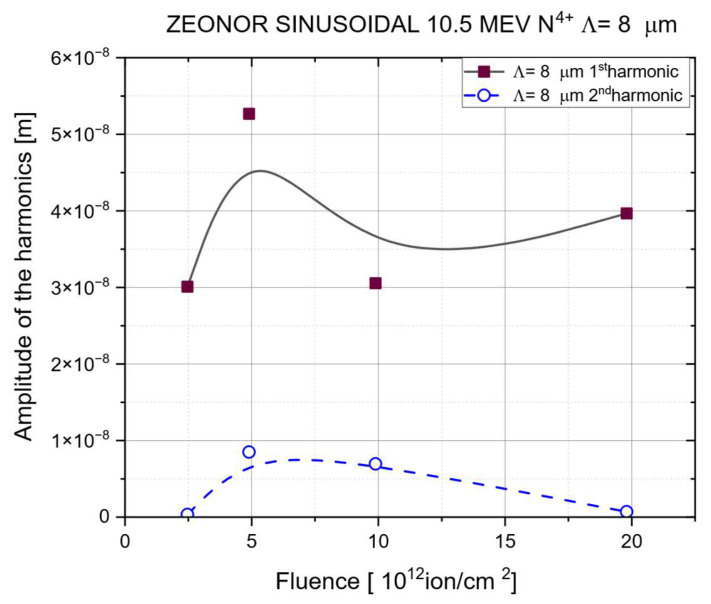
Amplitudes of the harmonics of the gratings implanted in ZEONOR™ by 10.5 MeV N^4+^ ions vs. implanted fluence. Λ = 8 μm. Black squares: measured values of the amplitude of the first harmonic. Blue circles: measured values of the amplitude of the second harmonic. Lines serve only to guide the eye.

**Figure 8:**
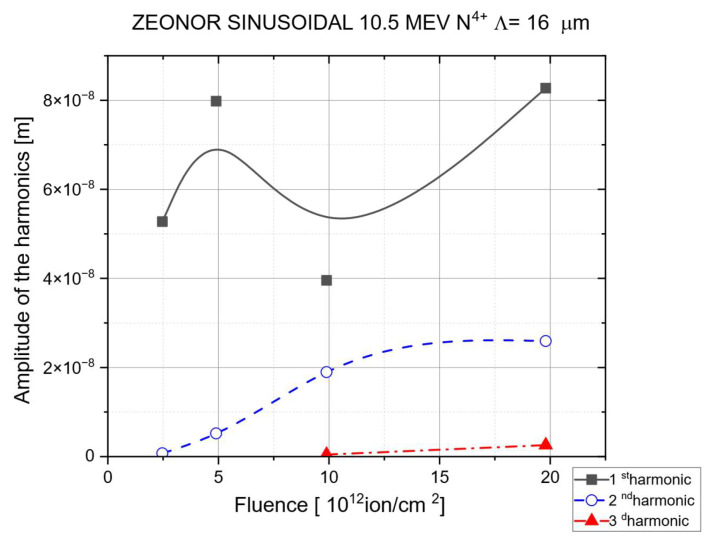
Amplitudes of the harmonics of the gratings implanted in ZEONOR™ by 10.5 MeV N^4+^ ions vs. implanted fluence. Λ = 16 μm. Black squares: measured values of the amplitude of the first harmonic. Blue circles: measured values of the amplitude of the second harmonic. Red triangles: measured values of the amplitude of the third harmonic. Lines serve only to guide the eye.

Peak-to-peak amplitudes of the harmonics of the grating profiles are shown in Figure 6, Figure 7 and Figure 8.

It can be observed in Figure 6 that the amplitude of the first harmonic of the profile of the Λ = 4 μm increases monotonically with the implanted fluence. Its maximum value is about 25 nm. The relatively low values of the amplitude of the first harmonic are due to the fact that they were written as binary gratings, i.e., each period of those gratings was written by a single pass of the microbeam and then with a scan without the ion beam. Thus, the amplitude of the gratings was limited by the width and profile of the ion microbeam. No higher harmonics could be measured.

Higher harmonics could be detected and measured in the case of gratings of grating constants of 8 and 16 μm (Figure 7 and Figure 8). The highest amplitudes of the first harmonic were 48 nm and 88 nm, respectively. However, the amplitude of the first harmonic did not increase monotonically with the implanted fluence. Instead, for both grating constants, there was a significant decrease in the amplitude around a fluence of 1·10^13^ ion/cm^2^. In the case of the Λ =16 μm grating, that drop may be attributed to a possible instrumental error during implantation, since the profile of the grating fabricated at a fluence of 1·10^13^ ion/cm^2^ has much wider crests than troughs, and crests consist of double peaks. In the case of the Λ = 8 μm grating implanted at a fluence of 1·10^13^ ion/cm^2^, no doubling of the crests could be observed, but the grating profile was much more irregular than that of the three other gratings of Λ = 8 μm. A sine function was fitted with R^2^ = 0.70 to the profile of that grating, while R^2^ was between 0.94 and 0.96 for the three other Λ = 8 μm gratings.

Diffraction efficiencies of all the optical gratings were measured at λ = 405 and 640 nm, using diode lasers. Measurements were performed in “s” polarization configuration, i.e., with the polarization of the laser beam perpendicular to the plane of incidence. Net diffraction efficiencies were calculated by correcting for reflection and absorption losses. The highest measured first-order and higher-order diffraction efficiencies at both wavelengths are presented in Table 1. It can be seen that the highest first-order diffraction efficiencies are close to the theoretical maximum of 0.33, predicted by the Raman-Nath theory [35]. For a pure surface relief grating, the Raman-Nath equation [36,37] is the following:

η*_m_* = J_m_^2^ (π Δnd/λ) (1)

where λ is the wavelength, d is the peak-to-peak amplitude of the grating, η*_m_* is the diffraction efficiency in the mth order, J_m_ is the m-order Bessel function, and Δn is the difference between the refractive index of the grating material and the surrounding material.

Δn = n_grating_ − n_air_ (2)

Note that according to the Raman-Nath equations, diffraction efficiency does not depend on grating constant. Another important fact is that perfectly sinusoidal gratings also have significantly higher-order diffraction efficiencies, as can be seen in Table 1.

Nonlinearity of the grating profile can also be taken into account in the framework of the Raman-Nath theory.

Diffraction in the first order from higher harmonics contributes to diffraction in higher orders from the first harmonic. For example, first-order diffraction from the second harmonic propagates at the same angle as second-order diffraction from the first harmonic. This problem was discussed in detail in one of our previous articles on ion microbeam implanted gratings [33].

**Figure 9:**
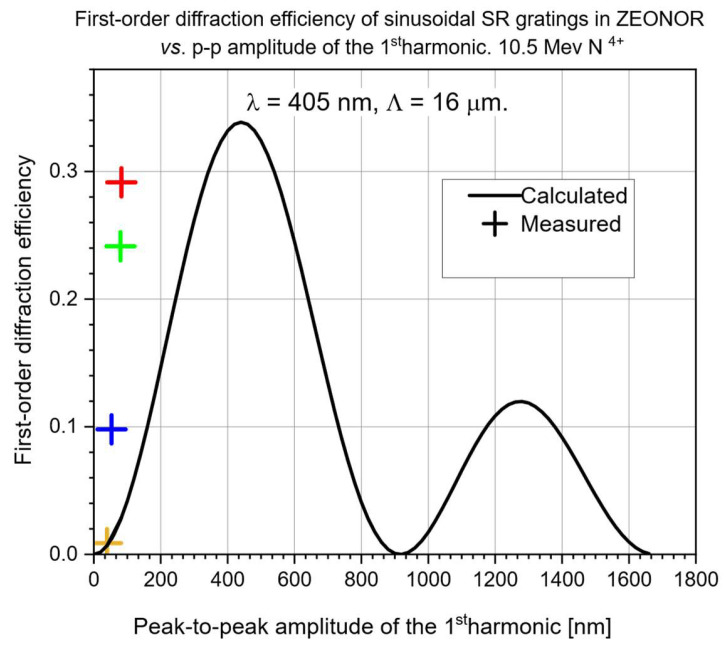
Calculated and measured first-order diffraction efficiency of surface relief optical gratings vs. the peak-to-peak amplitude of their first harmonic. Grating constant Λ = 16 μm. Black line: Calculated using Equation (1), with n_grating_ = 1.539. Symbols: Measured values. Measurements were performed at λ = 405 nm. Symbol size corresponds to the error of the measurement. Warmer colors of the symbols indicate higher implanted fluence. See Figure 8.

Measured and calculated first-order diffraction efficiency of the Λ =16 μm gratings, at λ = 405 nm, as a function of the amplitude of the first harmonic of the gratings is shown in Figure 9.

When using the refractive index of the unimplanted ZEONOR polymer at 405 nm, n_grating_ = 1.539, calculated values of the first-order diffraction efficiencies are much lower than the measured ones. To solve this problem, we hypothesized that ion implantation causes a significant increase in the refractive index of the ZEONOR copolymer. Very high refractive index changes in polymers due to ion beam implantations were reported by several authors. Biersack and Kallweit [38] implanted various ions, ranging from 300 keV protons to 200 KeV energy Xe^+^ ions into polymethylmethacrylate (PMMA) at various implanted fluences. They found that the refractive index changes could be attributed predominantly to the electronic interaction of the ions with the target material. The refractive index increase was proportional to the ion energy. The highest refractive index changes were obtained by 300 keV N^2+^ ions: Δn = 0.292, and 200 KeV Xe^+^ ions: Δn = 0.453. Fluences were kept fixed at F = 2·10^14^ ions/cm^2^, only 5 times higher than those used in the present work. Gupta et al. [39] implanted 100 keV N^+^ and Ar^+^ ions (separately) into PMMA and Polyethylene (PET) samples. The nitrogen ion implantation resulted in a very high refractive index increase in PMMA: Δn = 1.01 at F = 1·10^16^ ions/cm^2^ and Δn = 1.17 at F = 2·10^16^ ions/cm^2^. Sharma et al. [40] studied the changes in the physical properties of PMMA and PET samples when implanted by 50 keV B^+^ ions. They also found a giant refractive index increase in PMMA, Δn = 0.75 at relatively low fluence of F = 1·10^15^ ions/cm^2^, and Δn = 1.55 at F = 5·10^15^ ions/cm^2^. The maximum refractive index increase in the PET target, obtained with the ion beam of the same energy and the highest fluence, was lower, Δn = 0.6. The above refractive indices were measured at 600 nm. Full dispersions were measured, and refractive index changes were lower in the violet (around 400 nm).

Calculations of the diffraction efficiency of the gratings were also performed by using an increased index of refraction of the implanted ZEONOR material. It was found that setting n_grating_ = 2.539 (Δn = 1.000) would approximate well the measured values of the first-order diffraction efficiency.

Note that calculated diffraction efficiencies corresponded well to the measured ones in the case of ion microbeam-written surface relief transmission gratings in crystals and glasses [32,33].

## 4. Conclusions

A method for direct writing of micro- and nanostructures in a cyclic olefin polymer was proposed and demonstrated. Surface relief transmission optical gratings of quasi-sinusoidal profile and grating constants down to 4 µm were written in the sample with a 10.5 MeV energy N^4+^ ion microbeam at fluence ranges of 2.5–20·10^12^ ion/cm^2^. The measured highest first-order diffraction efficiencies were close to the theoretical maximum. The present fabrication method has some clear advantages over other microfabrication methods. As discussed in the Introduction, the majority of the other microfabrication methods involve various fabrication steps, such as chemical development. Standard microelectronic technology has various processing steps. Laser-based microfabrication methods have inherent lateral and depth resolution limitations due to the diffraction of light. The present fabrication method is a one-step process. It creates surface relief microstructures with predefined height profiles. Since the required fluences are very low, a large number of ion beam implanted devices can be fabricated during one session. The disadvantage of the proposed method is that the use of a particle accelerator is necessary. On the other hand, the advances in the capabilities of the accelerators allow for the reduction in the resolution limit of the proposed method. Ion nanobeam or ion nanoprobe facilities are becoming widely available [41,42,43,44]. Depending on the ion species, its energy and the nanobeam forming magnetic device, beam sizes down to 200 nm were achieved. Thus, surface relief optical gratings performing beam coupling at visible wavelengths can also be fabricated in ZEONOR™ and similar polymer materials for sensors. Besides gratings, fabrication of any kind of surface structure with lateral resolution of a few hundred nanometers can be envisaged using this technique. The fabricated samples could be used either directly or as masters for hot embossing.

The gratings were fabricated in 2022. Long-term stability tests were performed on the structures over a period of 2 years. Meanwhile, the samples were stored at room temperature and normal relative humidity. Diffraction efficiencies were repeatedly measured during two years, and no detectable changes were observed. Since the ion microbeam-implanted gratings were intended for biomedical sensing applications, no thermal or mechanical stress tests were performed. Evidently, no structure fabricated in this polymer is meant to operate above its glass temperature, i.e., 136 °C. In our earlier research, we found that the microhardness of the implanted structures in ZEONOR also remained stable [30].

## Figures and Tables

**Table 1:** Highest measured diffraction efficiencies, η_1max_, η_2max_ and η_3max_, for all three grating constants and the two wavelengths.

Grating Constant Λ [µm]	η_1max_ at λ = 405 nm	η_2max_ at λ = 405 nm	η_3max_ at λ = 405 nm	η_1max_ at λ = 640 nm	η_2max_ at λ = 640 nm	η_3max_ at λ = 640 nm
4	0.06	0.05	-	0.06	0.02	-
8	0.27	0.16	-	0.25	0.14	-
16	0.29	0.20	0.16	0.25	0.14	0.05

## Data Availability

Additional data and materials are available at request from the Corresponding author.
